# Phytochemical analysis reveals an antioxidant defense response in *Lonicera japonica* to cadmium-induced oxidative stress

**DOI:** 10.1038/s41598-022-10912-7

**Published:** 2022-04-27

**Authors:** Chengcheng Li, Yi Tang, Fengwu Gu, Xiaoqian Wang, Wei Yang, Yang Han, Yanan Ruan

**Affiliations:** 1 School of Life Science, Liaoning University, Shenyang, 110036 China; 2 Key Laboratory of Pollution Ecology and Environment Engineering, Institute of Applied Ecology, Chinese Academy of Sciences, Shenyang, 110016 China

**Keywords:** Plant physiology, Plant stress responses

## Abstract

Cadmium (Cd), though potentially beneficial at lower levels to some plant species, at higher levels is a toxic metal that is detrimental to plant growth and development. Cd is also a carcinogen to humans and other contaminated plant consumers, affecting the kidneys and reducing bone strength. In this study we investigated responses of growth, chlorophyll content, reactive oxygen species levels, and antioxidant responses to Cd in honeysuckle leaves (*Lonicera japonica* Thunb.), a potential Cd hyperaccumulator. Results indicated that plant height, dry weight, leaf area, and chlorophyll content increased when honeysuckle was exposed to 10 mg kg^−1^ or 30 mg kg^−1^ Cd (low concentration). However, in response to 150 mg kg^−1^ or 200 mg kg^−1^ Cd (high concentration) these growth parameters and chlorophyll content significantly decreased relative to untreated control plant groups. Higher levels of superoxide radical (O_2_^·−^) and hydrogen peroxide (H_2_O_2_) were observed in high concentration Cd groups. The activities of ascorbate peroxidase (APX), monodehydroascorbate reductase (MDHAR), dehydroascorbate reductase (DHAR), and glutathione reductase were enhanced with exposure to increasing levels of Cd. Additionally, the Ascorbate–Glutathione (AsA–GSH) cycle was activated for the removal of H_2_O_2_ in honeysuckle in response to elevated Cd. The Pearson correlation analysis, a redundancy analysis, and a permutation test indicated that proline and APX were dominant antioxidants for removing O_2_^·−^ and H_2_O_2_. The antioxidants GSH and non-protein thiols (NPTs) also increased as the concentration of Cd increased.

## Introduction

The ecological disruption caused by heavy metal soil contamination has been growing due to urbanization and industrialization as well as the increase in soil Cd content produced through commonly used phosphate soil fertilization^1^. Cd is the most common toxic contaminant found in soils^2,3^. It is readily absorbed by plant roots, transported to above-ground tissues, and absorbed by higher organisms when the contaminated plants are ingested even when the contaminant level is below the phytotoxicity threshold^4,5^.

Cd accumulation reduces growth and negatively affects metabolic factors in plants, thus impacting their basic developmental, physiological, and biochemical processes^6,7^. Research indicates that Cd alters photosynthesis, damages the internal structure of chloroplasts, inhibits the biosynthesis of chlorophyll, which as a result decreases chlorophyll content and causes leaf chlorosis^8,9^. Excessive Cd also has a negative impact on plant water relations which induces a water deficit, alters ion homeostasis, inhibits nutrient uptake of essential minerals such as Fe and Ca, all of which have a negative effect on plant growth^10–12^. Therefore, high levels of Cd in plants result in wilting and eventually in plant mortality^13,14^.

Cd is not a redox metal. It cannot directly participate in Fenton and Haber–Weiss reactions to produce reactive oxygen species (ROS)^15,16^. However, Cd impairs electron transport in mitochondria and chloroplasts, alters enzyme activity, and induces the production of ROS^17^. Excessive production and accumulation of ROS, such as superoxide anion (O_2_^·−^) and hydrogen peroxide (H_2_O_2_), alter the redox status of cells, resulting in oxidative injury, evidenced as an increase in ion leakage, lipid peroxidation, and DNA-strand cleavage^18^. Some plants have evolved a defense response against Cd and ROS accumulation, which includes activation of an antioxidant system and the production of osmoprotectants^12,19^. Enzymatic antioxidants include superoxide dismutase (SOD), catalase (CAT), ascorbate peroxidase (APX), monodehydroascorbate reductase (MDHAR), dehydroascorbate reductase (DHAR), glutathione reductase (GR), and glutathione-S-transferase (GST), while non-enzymatic antioxidants include molecules such as ascorbic acid (AsA) and glutathione (GSH)^20^. Enzymatic and non-enzymatic antioxidants are associated with the ascorbate–glutathione pathway which is one of the effective ways to scavenge H_2_O_2_ in plant cells^21^. AsA acts as an electron donor to process H_2_O_2_ into water and oxygen through APX catalysis reducing oxidative damage. In addition, DHAR and MDHAR are responsible for AsA regeneration^22^. GSH and NADPH also act as electron donors and are involved in H_2_O_2_ degradation^22^.

Glutathione can indirectly scavenge Cd-induced ROS. The change in the ratio of GSH and its oxidized form (GSSG) acts as a redox pair to modulate the signaling of antioxidant mechanisms in cells^20,23^. When the GSH/GSSG ratio is balanced, plants can reduce or eliminate the oxidative stress caused by Cd. Once the balance is disturbed as a result of high levels of ROS, plants will experience oxidative damage^24^. NPTs are molecular compounds in plant cells such as cysteine (Cys), GSH, phytochelatins (PCs), metallothioneins (MTs)^25^. These small molecules combine with free Cd to form complexes such as Cd-GSH and Cd-PC. The complexes are then compartmentalized in vacuoles, and thus no longer participate in Cd toxic reactions^26,27^. Proline, a signaling molecule, is an important osmoprotectant and antioxidant^28^ which functions to maintain the redox balance in cells acting as a free radical scavenger, metal chelator, cell membrane stabilizer, and activator of the ROS detoxification pathway^28–30^. The accumulation of proline was shown to be related to Cd-induced iron deficiency and the inhibition of electron transport activity^31,32^.

Honeysuckle (*Lonicera japonica* Thunb.) is a twining, semi-evergreen vine, distributed widely in temperate and tropical regions^33,34^. It is a popular landscape plant with high environmental adaptability^35^. Honeysuckle has a high Cd tolerance and a strong tendency to accumulate Cd. Its shoot Cd concentration reached 286.12 μg g^−1^ dry weight when exposed to 25 mg L^−1^ Cd for 20 days^36^. Honeysuckle is regarded as a potential Cd hyperaccumulator^36^, however, the biological and chemical mechanisms of Cd enrichment have not been sufficiently revealed in previous studies. In the present study, the morphological and physiological responses of honeysuckle to elevated levels of Cd were explored. The non-enzymatic antioxidant contents and antioxidant enzyme activities were monitored along with ROS levels in honeysuckle in response to various levels of Cd. Redundancy analysis (RDA) and a permutation test were used to identify the crucial components of the antioxidant system.

## Results

### Effects of Cd levels on plant growth and chlorophyll content

Several growth parameters in honeysuckle cuttings were measured to determine their response to different levels of Cd in the soil including plant height, dry weight, and leaf area. Relative to the control, 10 and 30 mg kg^−1^ Cd promoted honeysuckle growth as measured by plant height, dry weight, and leaf area, all of which were significantly greater (*P* < 0.05) after 90 days of Cd-exposure. In soils containing 80 mg kg^−1^ Cd, plant height and leaf area were not significantly different (*P* > 0.05) from the control. In contrast, both plant height and leaf area were significantly lower (*P* < 0.05) in cuttings exposed to 150 and 200 mg kg^−1^ Cd (Table 1).

**Table 1:** Effects of different concentrations of cadmium on growth parameters and chlorophyll content in *Lonicera japonica* Thunb. after 90 days.

Cd concentration (mg kg^−1^)	Height (cm)	Dry weight (g)	Leaf area (cm^2^)	Chlorophyll *a* (mg g^−1^ FW)	Chlorophyll *b* (mg g^−1^ FW)	Total chlorophyll (mg g^−1^ FW)
0	44.67 ± 0.58 b	6.74 ± 0.10 d	13.20 ± 0.13 b	6.50 ± 0.05 d	2.45 ± 0.12 d	8.95 ± 0.13 d
10	46.67 ± 0.58 a	8.69 ± 0.11 b	14.18 ± 0.60 a	7.69 ± 0.12 c	3.27 ± 0.41 ab	10.95 ± 0.29 c
30	47.33 ± 1.15 a	11.31 ± 0.50 a	14.63 ± 0.23 a	10.15 ± 0.02 a	3.17 ± 0.16 abc	13.32 ± 0.19 a
80	43.83 ± 0.76 b	7.09 ± 0.18 c	13.13 ± 0.34 b	8.56 ± 0.17 b	2.75 ± 0.11 cd	11.32 ± 0.15 b
150	41.33 ± 1.53 c	6.54 ± 0.14 d	11.32 ± 0.14 c	5.51 ± 0.15 e	3.50 ± 0.34 a	9.01 ± 0.18 d
200	40.83 ± 0.29 c	5.79 ± 0.18 e	11.25 ± 0.23 c	5.43 ± 0.12 e	2.94 ± 0.07 bc	8.37 ± 0.18 e

Different letters indicate a significant difference (*P* < 0.05) based on Duncan’s multiple range test at 5% level. Values are the mean ± SD (n = 3).

The contents of chlorophyll *a*, chlorophyll *b* and total chlorophyll increased significantly (*P* < 0.05) within 10, 30 mg kg^−1^ Cd after 90 days of Cd-exposure. The chlorophyll *a* content decreased significantly in leaves of cuttings from plants exposed to 150 mg kg^−1^ Cd (*P* < 0.05), In soils containing 200 mg kg^−1^ Cd, the content of chlorophyll *a* and total chlorophyll decreased significantly (*P* < 0.05) (Table 1).

### Effects of Cd levels on ROS levels

**Figure 1:**
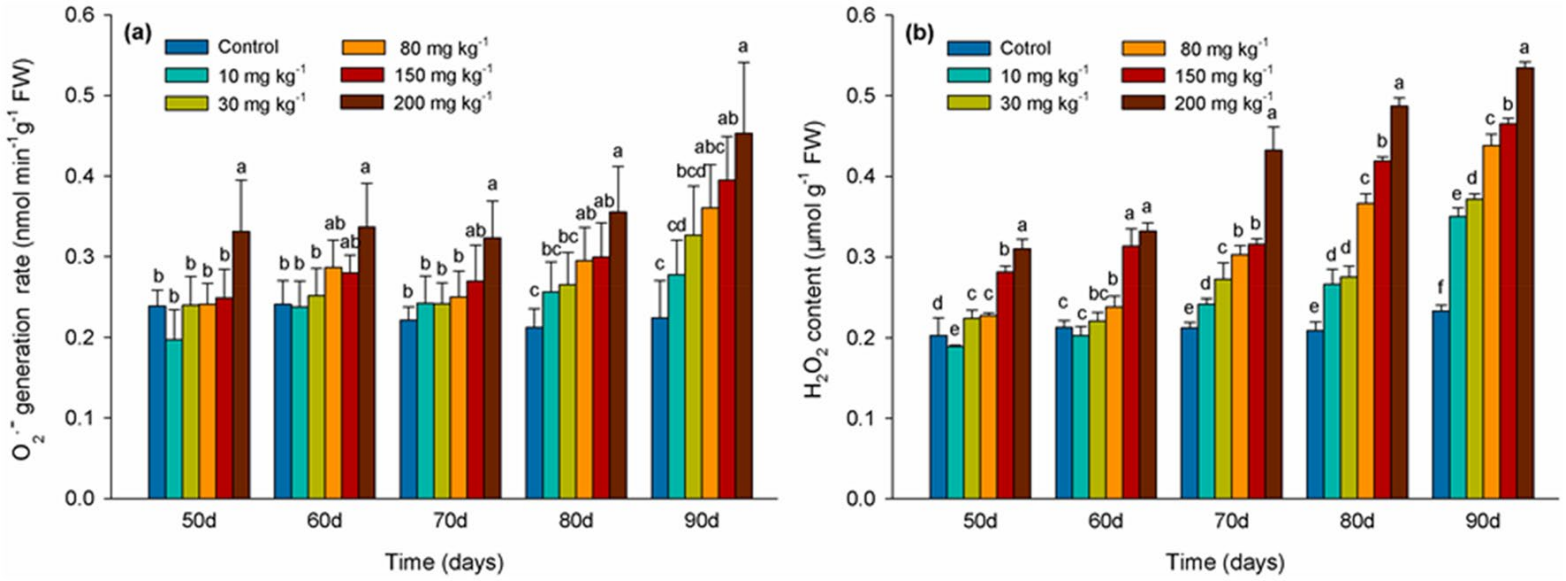
Rate of superoxide radical (O_2_^·−^) generation (**a**) and hydrogen peroxide (H_2_O_2_) levels (**b**) in leaves of *Lonicera japonica* Thunb. cuttings exposed to different concentrations of Cd. Different letters indicate a significant difference (*P* < 0.05) based on Duncan’s multiple range test at 5% level. Values are the mean ± SD (n = 3).

The rate of O_2_^·−^ generation in honeysuckle leaves compared to the control was not significantly different (*P* > 0.05) within the 10, 30, 80 mg kg^−1^ Cd treated samples before the 80 days of Cd exposure. In contrast, the rate of O_2_^·−^ generation was significantly higher in leaves of cuttings treated with 200 mg kg^−1^ Cd than that in control (Fig. 1a). H_2_O_2_ levels were significantly higher (*P* < 0.05) than in the control during the entire duration of the experiment in leaves of cuttings from plants exposed to 80, 150, and 200 mg kg^−1^ Cd. Notably, H_2_O_2_ levels were markedly increased (*P* < 0.05) at 90 days in leaves of cuttings exposed to all concentrations of Cd (Fig. 1b).

### Effects of Cd levels on antioxidant enzyme activity

**Figure 2:**
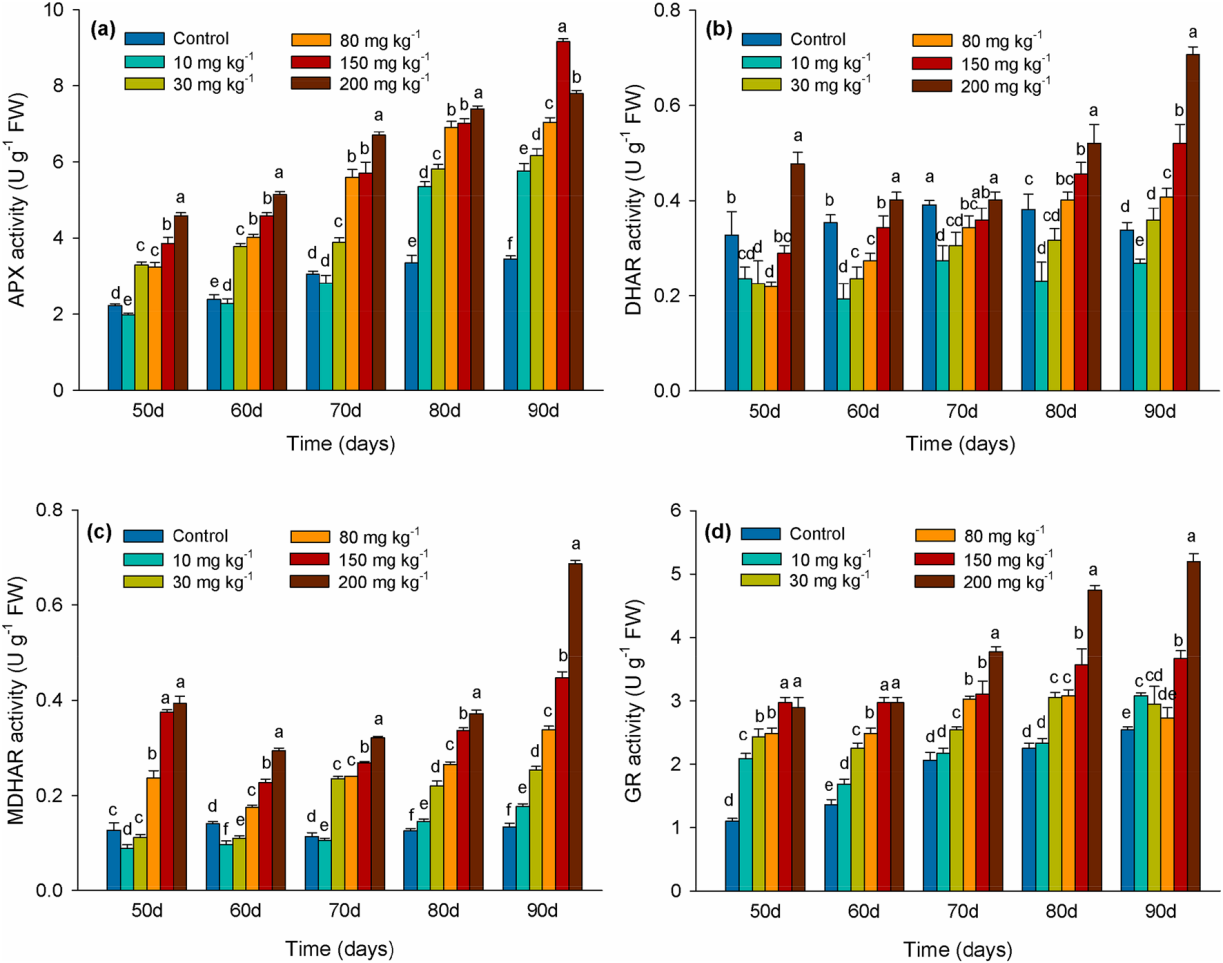
APX (ascorbate peroxidase) (**a**), DHAR (dehydroascorbate reductase) (**b**), MDHAR (monodehydroascorbate reductase) (**c**) and GR (glutathione reductase) (**d**) activities in leaves of *Lonicera japonica* Thunb. cuttings exposed to different concentrations of Cd. Different letters indicate a significant difference (*P* < 0.05) based on Duncan’s multiple range test at 5% level. Values are the mean ± SD (n = 3).

APX activity compared to the control decreased by 11% by day 50 in leaves of cuttings exposed to 10 mg kg^−1^ Cd. In sharp contrast, it was significantly higher than that in control after day 80. APX activity steadily increased in leaves of cuttings exposed to 30, 50, 80, 150, or 200 mg kg^−1^ Cd over the entire duration of the experiment (Fig. 2a). During the duration of the experiment, DHAR activity in leaves exposed to 10 mg kg^−1^ Cd was significantly lower (*P* < 0.05) than in the control group. In contrast, DHAR activity was significantly increased (*P* < 0.05) compared to the control group leaves in response to the 150 mg kg^−1^ Cd treatment (Fig. 2b). MDHAR activity consistently maintained an increased level compared to the control in leaves of cuttings exposed to 80, 150 and 200 mg kg^−1^. Prior to day 70, MDHAR activity was raised in leaves of cuttings exposed to 10 and 30 mg kg^−1^ Cd (Fig. 2c). GR activity exhibited an increasing trend over the entire duration of the experiment and reached its highest level in leaves of cuttings treated with 200 mg kg^−1^ Cd at day 90 (Fig. 2d).

### Effects of Cd levels on GSH pool and NPTs content

**Figure 3:**
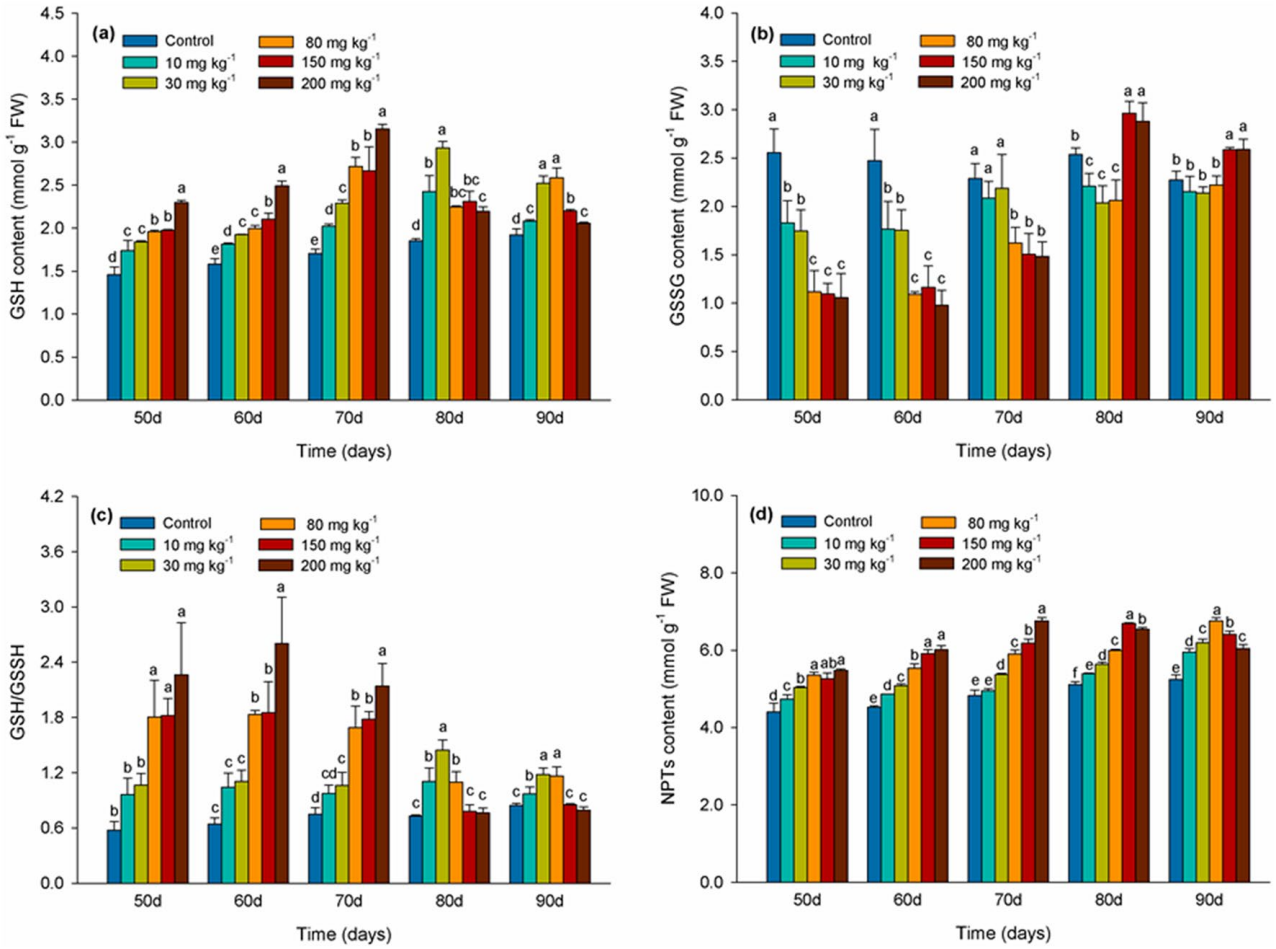
GSH (reduced glutathione) (**a**), GSSG (oxidized glutathione) (**b**), GSH/GSSG (**c**) and NPTs (non-protein thiols) (**d**) in leaves of *Lonicera japonica* Thunb. cuttings exposed to different concentrations of cadmium. Different letters indicate a significant difference (*P* < 0.05) based on Duncan’s multiple range test at 5% level. Values are the mean ± SD (n = 3).

As illustrated in Fig. 3a, the level of GSH significantly increased over time compared with the control in response to all Cd treatments through the 70 days. GSH levels reached a maximum on day 70 in response to the 80, 150, and 200 mg kg^−1^ Cd treatments, increasing by 69%, 65%, and 99%, respectively. Glutathione levels gradually decreased after day 80 in leaves of cuttings exposed to 150 and 200 mg kg^−1^ Cd. The level of GSSG was significantly higher (*P* < 0.05) than in the control in the 150 and 200 mg kg^−1^ Cd treatments after 80 days (Fig. 3b). The ratio of GSH/GSSG decreased after 80 days in leaves treated with 150 and 200 mg kg^−1^ Cd resulting in no significant (*P* > 0.05) difference with the control at this time interval (Fig. 3c). As indicated in Fig. 3d, NPTs levels increased in response to the different Cd treatments and were significantly higher than in the control (*P* < 0.05). NPTs content was highest at day 90 in the 80 mg kg^−1^ Cd treatment, but was much lower in the 150 and 200 mg kg^−1^ Cd treatments (Fig. 3d).

### Effects of Cd levels on proline content

**Figure 4:**
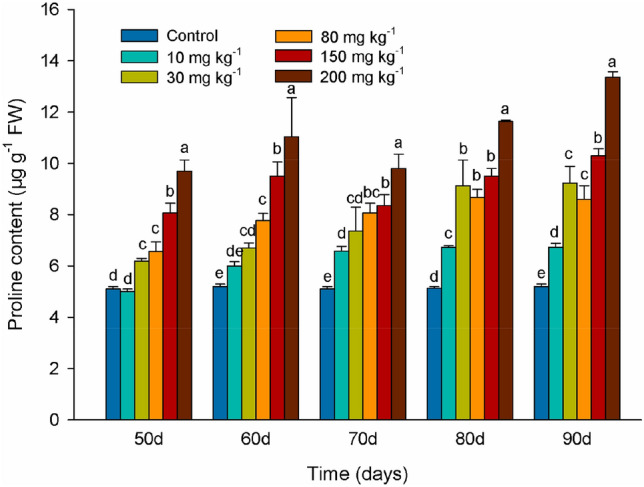
Proline levels in leaves of *Lonicera japonica* Thunb. cuttings exposed to different concentrations of Cd. Different letters indicate a significant difference (*P* < 0.05) based on Duncan’s multiple range test at 5% level. Values are the mean ± SD (n = 3).

The proline content in leaves of the 10 mg kg^−1^ Cd treatment markedly increased after 70 days suggesting an adaptation to higher Cd saturation levels accumulated over time. Proline content in leaves continually increased over the duration of the experiment in leaves of cuttings from plants exposed to 30, 50, 80, 150, and 200 mg kg^−1^ Cd. On day 90, proline content was 98% and 157% higher in leaves exposed to 150, and 200 mg kg^−1^ Cd respectively than in control (Fig. 4).

### Correlation between Cd levels and measurement indexes

**Figure 5:**
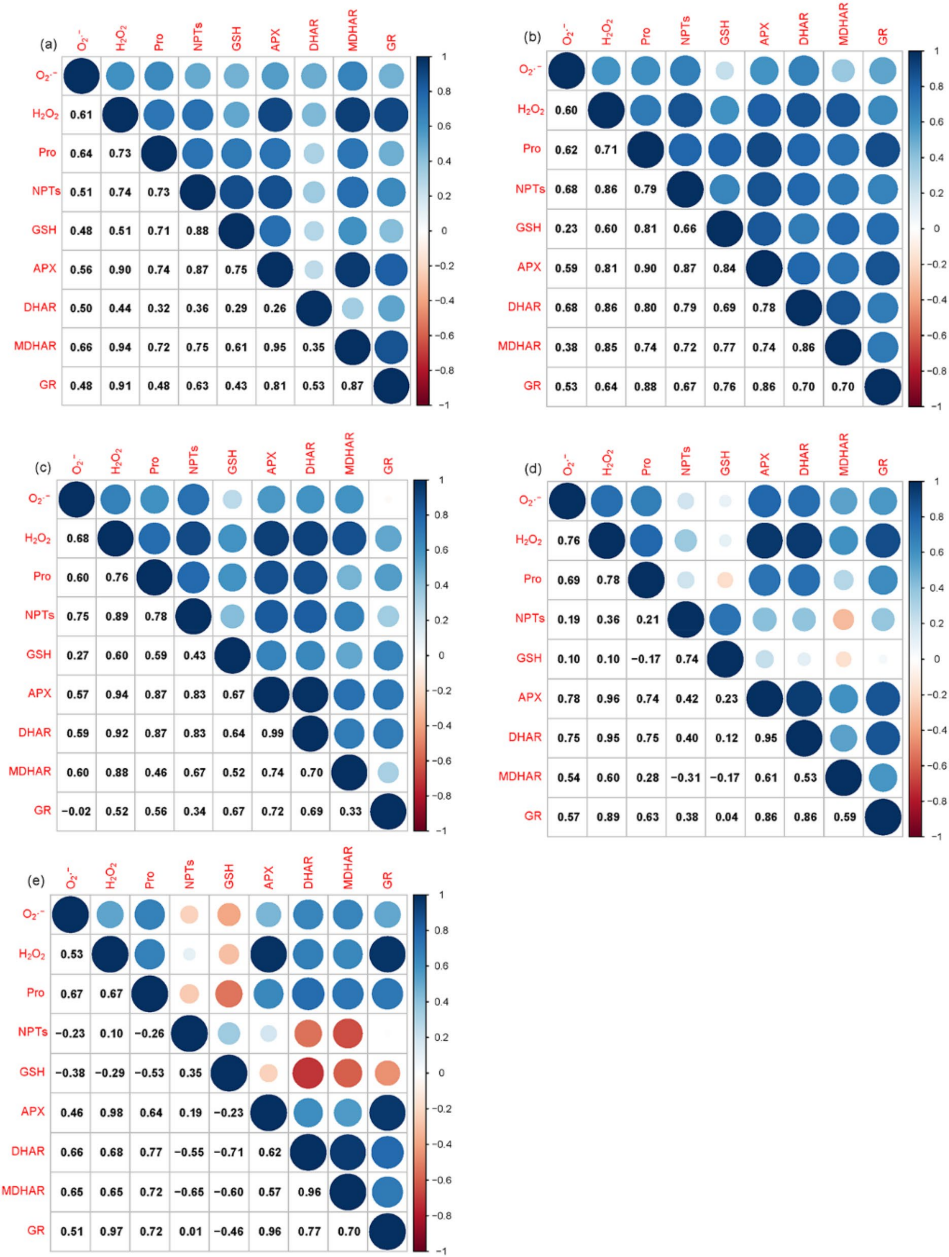
The Pearson correlation coefficients among the measured variables in leaves of *Lonicera japonica* Thunb. cuttings exposed to different Cd treatments. (**a**) 10 mg kg^−1^ Cd; (**b**) 30 mg kg^−1^ Cd; (**c**) 80 mg kg^−1^ Cd; (**d**) 150 mg kg^−1^ Cd; (**e**) 200 mg kg^−1^ Cd.

As shown in Fig. 5, APX and GR had a significant positive correlation with H_2_O_2_ (*P* < 0.05, r_APX_ = 0.90, 0.81, 0.94, 0.96 and 0.98, r_GR_ = 0.91, 0.64, 0.52, 0.89 and 0.97, respectively) in the 10, 30, 80, 150 and 200 mg kg^−1^ Cd treatment. MDHAR had a significant positive correlation with H_2_O_2_ (*P* < 0.05, r_MDHAR_ = 0.94, 0.85, 0.88, and 0.60, respectively) in 10, 30, 80, and 150 mg kg^−1^ Cd exposure. GSH had a significant positive correlation with H_2_O_2_ (*P* < 0.05, r_GSH_ = 0.51, and 0.60, respectively) in 10 and 30 mg kg^−1^ Cd exposure. NPTs had a significant positive correlation with H_2_O_2_ (*P* < 0.05, r_NPTs_ = 0.74, 0.86 and 0.89, respectively) in 10, 30, and 80 mg kg^−1^ Cd exposure. Proline had a significant positive correlation with H_2_O_2_ (*P* < 0.05, r_Pro_ = 0.71, 0.76 and 0.78, respectively) in 30, 80, 150 mg kg^−1^ Cd exposure. O_2_^·−^ had a significant positive correlation with proline, NPTs, APX, and DHAR (*P* < 0.05, r = 0.60, 0.75, 0.57, and 0.59, respectively) in 80 mg kg^−1^ Cd exposure.

### RDA and permutation tests of the measurement indexes with Cd levels

**Figure 6:**
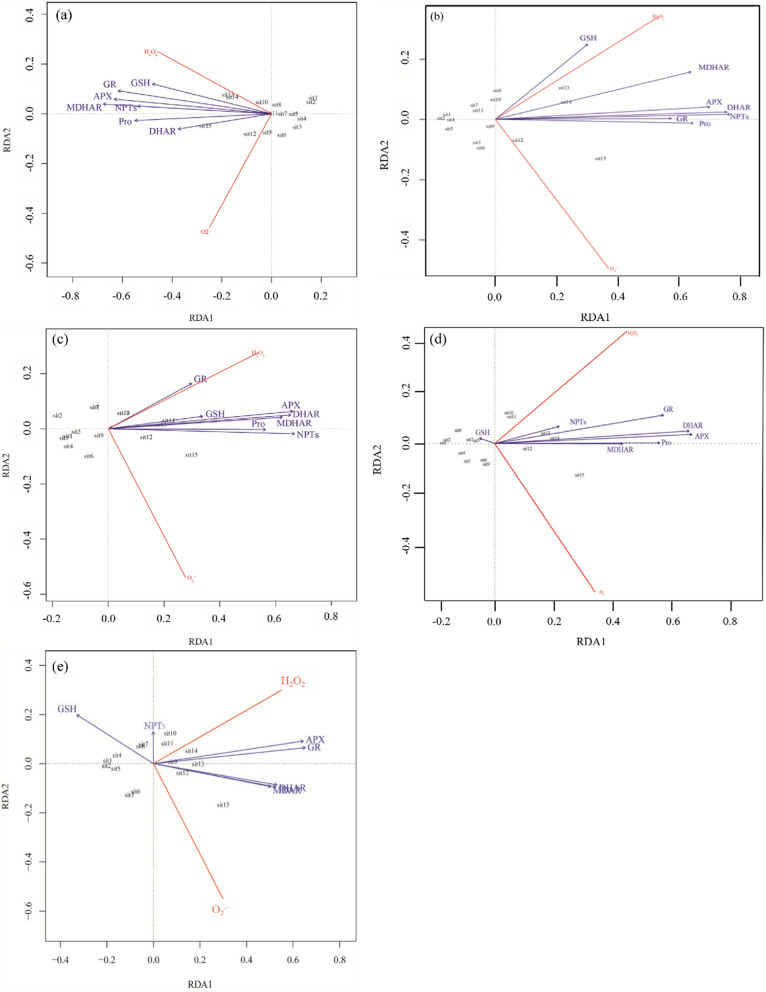
Triplot of the redundancy analysis (RDA) of H_2_O_2_ and O_2_^·−^ generation rate in leaves of *Lonicera japonica* Thunb. cuttings exposed to different Cd treatments. (**a**) 10 mg kg^−1^ Cd; (**b**) 30 mg kg^−1^ Cd; (**c**) 80 mg kg^−1^ Cd; (**d**) 150 mg kg^−1^ Cd; (**e**) 200 mg kg^−1^ Cd.

As showed in Fig. 6, proline, GSH, NPTs, APX, DHAR, MDHAR, and GR were positively correlated with H_2_O_2_ and O_2_^·−^ generation rate in leaves of honeysuckle cuttings exposed to 10 mg kg^−1^ Cd (Fig. 6a). Notably, Proline, GSH, and APX were significant predictor variables at the 0.05 probability level (*F*_Pro_ = 25.2, *F*_GSH_ = 4.56, *F*_APX_ = 6.54). Proline, GSH, NPTs, APX, DHAR, MDHAR, and GR were positively correlated with H_2_O_2_ and O_2_^·−^ in leaves of honeysuckle cuttings exposed to 30 mg kg^−1^ Cd (Fig. 6b). Proline, GSH, and NPTs were significant predictor variables at the 0.05 probability level (*F*_Pro_ = 20.1, *F*_GSH_ = 4.44, *F*_NPTs_ = 6.54). Proline, GSH, NPTs, APX, DHAR, MDHAR, and GR were positively correlated with H_2_O_2_ and O_2_^·−^ in leaves of honeysuckle cuttings exposed to 80 mg kg^−1^ Cd (Fig. 6c). Proline, NPTs, APX, and MDHAR were significant predictor variables at the 0.05 probability level (*F*_Pro_ = 47.69, *F*_NPTs_ = 18.20, *F*_APX_ = 9.15, *F*_MDHAR_ = 6.93). Proline, NPTs, APX, DHAR, MDHAR, and GR were positively correlated with H_2_O_2_ and O_2_^·−^ in leaves of honeysuckle cuttings exposed to 150 mg kg^−1^ Cd. In contrast, GSH in the same treatment was negatively correlated with H_2_O_2_ and O_2_^·−^ (Fig. 6d). Proline and APX were significant predictor variables at the 0.05 probability level (*F*_Pro_ = 31.27, *F*_APX_ = 12.14). Proline, APX, DHAR, MDHAR, and GR were positively correlated with H_2_O_2_ and O_2_^·−^ in leaves of honeysuckle cuttings exposed to 200 mg kg^−1^ Cd. In the same treatment, GSH was negatively correlated with H_2_O_2_ and O_2_^·−^ (Fig. 6e). Proline and APX were significant predictor variables at the 0.05 probability level (*F*_Pro_ = 51.08, *F*_APX_ = 9.42).

## Discussion

Cd is a water-soluble element and is non-essential to plant growth^7^. Cd can bind to transporters used by plants to transfer certain essential elements^17^. This reduces the capacity of the plant to absorb, transport, and utilize essential elements and thus affects the growth of plants to varying degrees^10^. Reduction of these elements can be detrimental to the growth of the plant as well as reducing the nutrition provided to humans and livestock when consumed. This may due to shared root transporters through genetic expressions between Cd and Zn^2+^ transporter gene, and Fe^2+^ through transporter genes, which also transport Cd ions to the roots of common agricultural products such as rice causing micronutrient deficiency in supported populations^37^. In the current study, chlorophyll *a*, *b* and total chlorophyll content, plant height, dry weight, and leaf area increased in honeysuckle leaves of cuttings exposed to 10 and 30 mg kg^−1^ Cd. Our findings reinforce previous research showing that honeysuckle has a strong tolerance to moderate Cd exposure^36^. Cd has previously been reported to have a stimulating effect on plant growth at low concentrations^38^. In general, mild stress may stimulate plants to initiate a stress response that accelerates growth. The growth of cotton callus was stimulated by exposure to 0.55 mmol L^−1^ and 0.7 mmol L^−1^ (low concentration) Cd and inhibited by 1 mmol L^−1^ (high concentration) Cd^39^. Our experiment shows that honeysuckle has rapid biomass accumulation and acts as a Cd hyperaccumulator, which could be used to remediate Cd pollution levels. When plants are exposed to excessive amounts of Cd, reductions in plant growth, mineral nutrients, and biomass appear to be attributable to toxic effects of Cd^40^. In our study, the growth and chlorophyll content of honeysuckle decreased when the Cd concentration exceeded 80 mg kg^−1^, which was especially evident at 200 mg kg^−1^, where chlorophyll *a*, *b* and total chlorophyll contents were severely impacted (Table 1). Decrease in chlorophyll content may also be related to degradation of the chlorophyll structure caused by the accumulation of ROS in response to Cd stress^41^.

When plants are exposed to excessive levels of metals, they produce high levels of ROS, a phenomenon that is considered as one of the earliest biochemical changes exhibited by plants in response to metal induced stress^18^. The H_2_O_2_ content in leaves of honeysuckle increased when cuttings were exposed to increasing concentrations of Cd (Fig. 1). Low concentrations of Cd have been reported to induce low ROS levels that act as signal molecules in the induction of defense genes against Cd toxicity^42^. However, high concentrations of Cd induce high ROS levels generally causing a serious imbalance to occur in ROS synthesis and degradation. Plants are subjected to oxidative stress when high ROS levels are present posing a physiological challenge. In our study O_2_^·−^ and H_2_O_2_ levels in honeysuckle leaves increased significantly in response to the 150 and 200 mg kg^−1^ Cd treatments. Similar results were observed in a study of wheat roots showing that O_2_^·−^ generation rate and H_2_O_2_ content were also increased in response to elevated Cd exposure^43^. Elevated ROS levels can result in the inhibition of enzyme activity, protein oxidation^44,45^, and an inability to manage the higher levels of oxidative damage induced by ROS levels^43^.

Measuring SOD over the duration of the experiment did not provide statistically significant data. After repeated tests the decision was made to use the main antioxidant enzymes in the ascorbic acid glutathione cycle to illustrate the important role of antioxidant enzymes in resisting Cd stress. An important antioxidant system involved in Cd detoxification is the AsA-GSH cycle composed of several antioxidants such as GSH, AsA, and critical antioxidant enzymes APX, MDHAR, DHAR, and GR^46^. APX plays a vital role in the antioxidant defense response of plants by catalyzing the conversion of H_2_O_2_ to water at the expense of AsA^24^. In the present study APX activity was increased as the duration of Cd exposure increased and Cd concentration intensified. APX activity had a significant linear correlation with H_2_O_2_ content in all Cd-exposed treatment. This suggests that APX participates in detoxifying H_2_O_2_, and might be a crucial factor in eliminating ROS in elevated Cd stress. In the AsA-GSH cycle, AsA, as an electron donor of H_2_O_2_, produces dehydroascorbic acid (DHA) through the activity of APX and then converts it to AsA through the activity of DHAR when GSH, as a product of GR, is present as an electron donor^47^. In the present study, we also observed that MDHAR, DHAR and GR activity levels were elevated as exposure to levels of Cd increased (Fig. 2b–d). The enhanced activities of the AsA-GSH cycle may be attributed to the need to maintain a favorable redox status by maintaining sufficient levels of GSH and reduced AsA to overcome the physiological repercussions of oxidation^46^.

GSH functions to regulate H_2_O_2_ levels in plant cells and acts as an antioxidant, reducing oxidative stress caused by metal-induced ROS^48^. In our study, GSH content increased and the GSH/GSSG ratio was up-regulated in response to the 10 and 30 mg kg^−1^ Cd treatment. A lower level of ROS was also observed at this concentration of Cd (Fig. 1) which could be explained by the key role of GSH in scavenging of ROS. Change in the ratio of GSH/GSSG during the degradation of H_2_O_2_ plays an important role in some redox signaling pathways^49^. GSH content decreased at later testing periods in 150 and 200 mg kg^−1^ Cd treatment, while GSSG content continued to increase. Data shows that at 70 days glutathione GSH is highest (Fig. 3a), and the oxidized glutathione GSSG is decreasing (Fig. 3b). In addition, the level of hydrogen peroxide is continuing to rise (Fig. 1b), and the consumption of glutathione is low. During this period as the GSH/GSSG ratio decreased the ROS levels increased. A decrease in GSH in response to Cd stress suggests that the protective role of GSH against oxidative stress may be significantly reduced as Cd levels increase beyond a sustainable threshold^50^. NPTs molecules, including GSH, Cysteine, MTs (metallothioneins), and other related substances, contain a high percentage of cysteine sulfhydryl residues that play an important role in the detoxification of metals in plants^51^. In our study, the content of NPTs also increased significantly as the concentration of Cd increased, suggesting that NPTs might have participated in the detoxification of Cd (Fig. 3d) which also correlates with the study of Cd management in perennial ryegrass (*Lolium perenne* L.)^52^. The concentration of NPTs compounds also increased as the concentration of Cd increased in *Brassica pekinensis* and *B. chinensis*, which might have been due to the chelation of sulfhydryl compounds with Cd^53^.

In many plants, free proline accumulates in response to a wide range of biotic and abiotic stresses^54^. Proline has multiple functions in stress adaption^55^. Proline acts as an osmoprotectant and an antioxidant by scavenging hydroxyl radicals (OH^**.**^) and singlet oxygen (^1^O_2_) to alleviate ROS-induced cellular injury^56^. In the current study, proline content increased both as time of exposure to elevated Cd and as the concentration of Cd increased (Fig. 4). It is possible that an increase in the concentration of H_2_O_2_ and O_2_^·−^ induced free proline accumulation as the need to eliminate ROS increased in honeysuckle leaves.

Previous studies have demonstrated that Cd can induce an increase in the level of ROS in cells, whereas enzymatic and non-enzymatic antioxidants play an essential role in reducing excess ROS levels^20,57–59^. To expand on this knowledge, we analyzed the relationship between H_2_O_2_, O_2_^·−^, and antioxidants in the leaves of honeysuckle cuttings exposed to various concentrations of Cd by measuring specific antioxidant-related variables and determining their correlations using the RDA and permutation tests. Results indicated that when the content of H_2_O_2_ and O_2_^·−^ increased in honeysuckle leaves in response to increasing concentrations of Cd, proline levels also increased. A positive correlation between proline and H_2_O_2_ and O_2_^·−^ levels, and significant predictor variables were at the 0.05 probability level in 10, 30, 80, 150, and 200 mg kg^−1^ Cd exposure (Fig. 6). We confirmed that proline in honeysuckle leaves served as the dominant antioxidant in all Cd treatments, and accumulated proline acted as a protective response against oxidative stress^60^. Pearson correlation coefficients and permutation tests analysis (Fig. 6) indicated that the level of APX had a significant correlation with H_2_O_2_ content, in addition to proline. APX was an important predictor of oxidative stress in the 10, 80, 150 and 200 mg kg^−1^ Cd treatments. These factors suggested that APX, as an antioxidant enzyme, also plays an important role in scavenging H_2_O_2_ in honeysuckle leaves exposed to high concentration of Cd.

**Figure 7:**
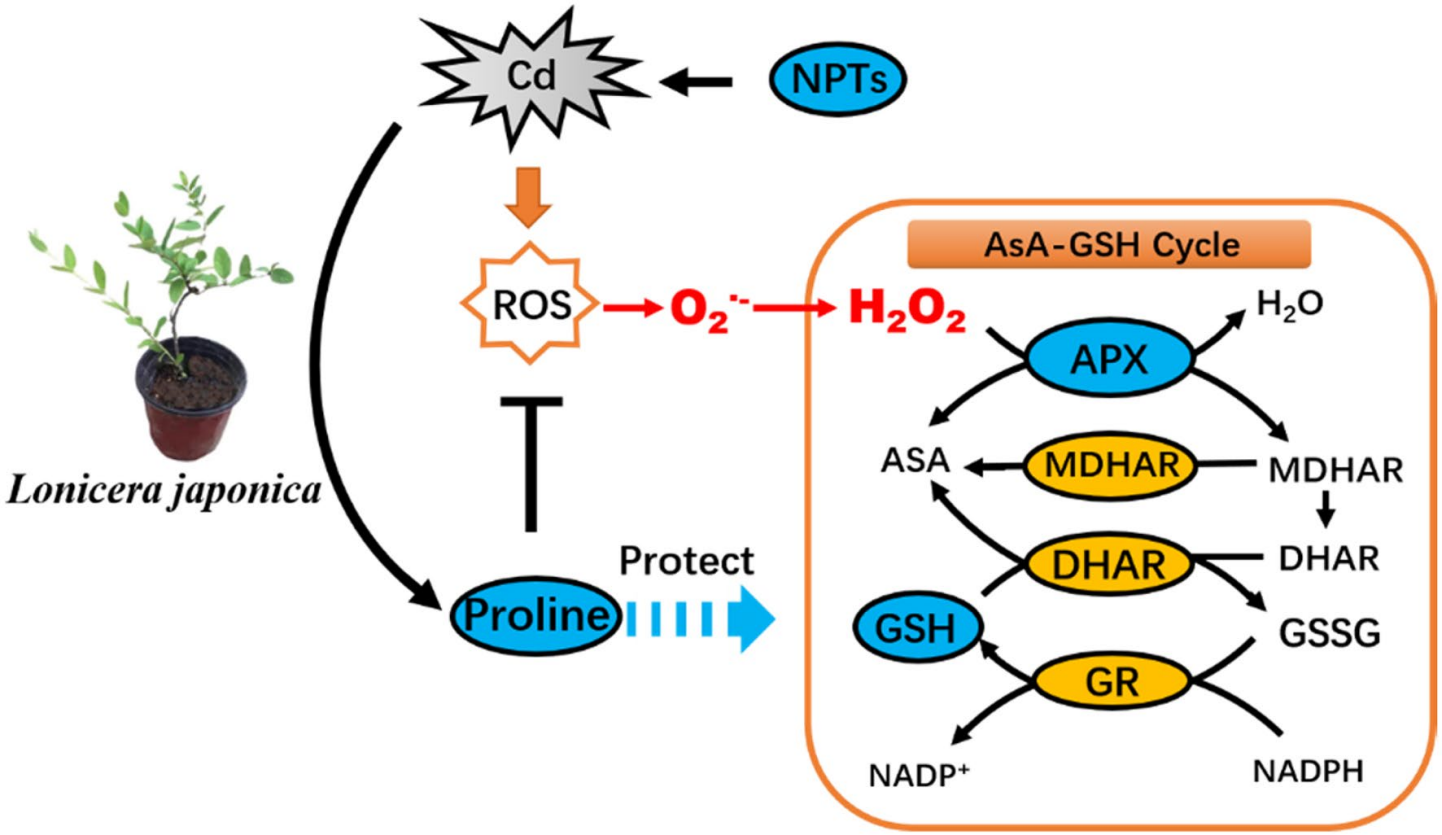
The antioxidant defense response in *Lonicera japonica* to Cd-induced oxidative stress.

GSH was correlated with H_2_O_2_ and O_2_^·−^, and significant predictor variables at the *P* < 0.05 probability level in honeysuckle leaves of cuttings exposed to 10 and 30 mg kg^−1^ Cd. This positive correlation, however decreased as Cd concentration increased, and was even a negative correlation in the 150 and 200 mg kg^−1^ Cd treatments. This response may occur at high concentrations of Cd when GSH mechanisms are overwhelmed and unable to act as an effective antioxidant. When GSH is consumed in higher quantities in plant cells, the increasing role of NPTs in scavenging ROS became increasingly evident. NPTs levels were significant predictor variables correlating with the content of H_2_O_2_ and O_2_^·−^ generation rate in 80 mg kg^−1^ Cd treatments. Compounds containing cysteine sulfhydryl residues including GSH played a major role in alleviating potential oxidative damage (Fig. 7).

## Materials and methods

### Plant material

Cuttings selected for this study were healthy, 1 year-old honeysuckle (*Lonicera japonica* Thunb.) from a representative strain cultivated at Liaoning University which continues to be used in additional research in the university's Department of life sciences. The cuttings were obtained in accordance with all relevant institutional, national, and international guidelines and legislation and all appropriate permissions and licenses were obtained for the specimens and materials. The cuttings were cultivated in sterilized sand for 10 weeks and then transplanted to a mixture of soil to sand (3:1). The soil was a brown topsoil (0–20 cm, pH 7.06), and was obtained from Liaoning University. The level of basic nutrients in the soil was 0.21 mg kg^−1^ calcium, 97.29 mg kg^−1^ nitrogen, 8.84 mg kg^−1^ phosphorus, 216.98 mg kg^−1^ potassium, and 1.50% organic matter. Air-dried soil samples were filtered through a 4.0 mm sieve for use in the experiment.

### Cd exposure

The experiments were conducted in a laboratory in Liaoning University 25/17 °C day/night temperature, 50–60% relative humidity, 16 h light/8 h dark) starting in March, 2017. Eighteen plastic pots (20 cm diameter × 30 cm height) containing 2.5 kg of air-dried, disinfected soil were prepared. A CdCl_2_·2.5H_2_O solution of different concentrations of Cd were randomly added to each pot. Six levels of Cd were administered to the pots, including 0 (control), 10, 30, 80, 150, and 200 Cd mg kg^−1^ soil (each treatment was replicated three times). The soil was regularly mixed and allowed to come to equilibrium over a period of 40 days. During this time, the soil was mixed and sprayed with water every week to maintain an 80% water content. Three honeysuckle cuttings with similar growth and height were planted in each pot after the 40 days equilibrium period. Leaves from the cuttings were harvested every ten days from the 50th through the 90th day after the cuttings were placed in the Cd-containing soils. Leaf samples were wrapped in tin foil, immediately frozen in liquid nitrogen, and stored at − 80 °C until subsequent analysis.

### Measurement of growth parameters

Height (cm) and leaf area (A, cm^2^) of plants were measured after 90 days of Cd exposure. At that time, all plants were collected. Roots were washed with distilled water, submerged in EDTA-Na_2_ for 20 min to balance ion levels, then cleaned with deionized water and immediately dried on filter paper. Lastly, the whole above-ground portion of the plant was placed in a 105 °C oven to a constant dry weight for the determination of biomass.

### Determination of chlorophyll content

Chlorophyll was extracted from 0.5 g fresh leaves with 80% acetone (centrifuging at 5000 rpm) and the absorbance of the supernatant was measured at 663 nm and 645 nm to determine the level of chlorophyll *a*, chlorophyll *b*, and total chlorophyll, respectively^61^.

### Determination of ROS levels

The rate of O_2_^·−^ generation in leaves was determined by the hydroxylamine hydrochloride method^62^ with minor modifications. Initially, 0.1 g leaves were ground in 3 ml 0.05 mol L^−1^ phosphate (K–P) buffer (pH 7.8), followed by centrifugation at 5000 rpm, 3 min at 4 °C. Subsequently, 0.5 ml supernatant was mixed with phosphate buffer (pH 7.8) and 1 mol L^−1^ hydroxylamine hydrochloride and incubated for 20 min at 25 °C. Then, 17 mmol L^−1^ p-aminobenzene sulfonic acid and 7 mmol L^−1^ 1-naphthylamine were added to the solution and absorbance was measured at 530 nm.

Hydrogen peroxide (H_2_O_2_) levels were determined in an extract prepared from 0.5 g leaves in 2.5 ml propanone, which was then centrifuged at 12,000 rpm for 10 min at 4 °C. The resulting supernatant was added to a mixture of 0.1 ml 5% Ti (SO_4_)_2_ (titanium sulphate) and 0.2 ml NH_3_ (ammonia), and then centrifuged at 10,000 rpm for 10 min at 4 °C. The resulting precipitate was dissolved in 2 mol L^−1^ H_2_SO_4_ and then re-centrifuged. The absorbance of the supernatant was measured at a wavelength of 415 nm using an ultraviolet spectrophotometer (UV-2100, UNICO, Shanghai, China)^63^.

### Determination of enzymatic and non-enzymatic antioxidant compounds

A total of 0.5 g of fresh leaves were ground in 3.5 ml 50 mmol L^−1^ phosphate buffer (K–P; pH 7.8) containing 1.0 mmol L^−1^ EDTA-Na_2_, 1.0 mmol L^−1^ ascorbate and 2% (v/v) polyvinylpyrrolidone (PVP), and 1.5 ml saturated ammonium sulfate. The mixture was then centrifuged at 5000 rpm for 10 min at 4 °C (GF16RXII, HITACHI, Tokyo, Japan). The resulting supernatant was used to measure enzyme activity.

Ascorbate peroxidase (APX; EC 1.11.1.11) activity: A 1 ml reaction mixture containing phosphate buffer (pH 7.0), 0.83 ml ascorbate, 0.13 ml H_2_O_2_, 0.04 ml crude enzyme was utilized. Ascorbate consumption was measured by the reduction in absorbance at 290 nm over 1 min. APX activity was calculated using an extinction coefficient of 2.8 (mmol L)^−1^ cm^−1^^64^.

Dehydroascorbate reductase (DHAR; EC 1.8.5.1) activity: A 1 ml reaction mixture containing 0.7 ml phosphate buffer, 0.1 ml reduced glutathione (GSH), 0.1 ml dehydroascorbate (DHA), and 0.1 ml crude enzyme extract was utilized. DHAR activity was calculated from the changes of absorbance at 265 nm over 1 min, using an extinction coefficient of 14 (mmol L)^−1^ cm^−1^^64^.

Monodehydroascorbate reductase (MDHAR; EC 1.6.5.4) activity: A 1 ml reaction mixture containing phosphate buffer (pH 7.6), 0.9 ml ascorbate, 0.04 ml ascorbate oxidase, 0.03 ml NADPH, and 0.03 ml of crude enzyme extract was utilized. MDHAR activity was determined by measuring the consumption of NADPH as indicated by the change in absorbance at 340 nm over 1 min, using an extinction coefficient of 6.2 (mmol L)^−1^ cm^−1^^65^.

Glutathione reductase (GR; EC 1.6.4.2) activity: A 1 ml reaction mixture containing 0.86 ml oxidized glutathione (GSSG), 0.1 ml NADPH, and 0.04 ml of crude enzyme extract was utilized. GR activity was calculated from the change in absorbance at 340 nm over 1 min, using an extinction coefficient of 2.8 (mmol L)^−1^ cm^−1^^64^.

GSH was determined according to the method of Yu et al.^66^ with a few modifications. Fresh leaves (0.15 g) were extracted with 1.75 ml 5% (w/v) sulfosalicylic acid, followed by centrifugation at 12,000×*g* for 4 min at 4 °C. The supernatant was used to determine the reduced and total glutathione content. Initialy, a 0.6 ml 0.1 mol L^−1^ phosphate buffer (K–P; pH 7.0; containing 0.5 mol L^−1^ EDTA) and 50 μl 3 mmol L^−1^ DTNB (5,5′-dithiobis-(2-nitrobenzoic acid)) was added to the supernatant and the content of reduced glutathione was determined by measuring the absorbance of the solution at 412 nm for 5 min. Then, 0.5 ml phosphate buffer, 50 μl DTNB, 0.1 ml NADPH (0.4 mmol L^−1^), and 2 μl GR were added to the supernatant and the solution was incubated for 20 min. Total glutathione was determined by measuring absorbance at 412 nm. Oxidized glutathione (GSSG) content was determined based on the difference between the values of total glutathione content and reduced glutathione content (GSSG). The obtained values were used to determine the GSH/GSSG ratio.

### Determination of non-protein thiols (NPTs) and proline content

NPTs were measured as previously described by Sharma et al.^67^. Initially, 0.1 g of sample was ground in 5 ml 1 mol L^−1^ HCl and 1 mol L^−1^ EDTA, and centrifuged at 10,000 rpm for 3 min at 4 °C. The supernatant was then added to 0.5 ml phosphate buffer (pH 7.8), and 0.5 ml 6 mmol L^−1^ DTNB, N levels were determined by measuring the change in absorbance at 412 nm.

Proline content was determined using the acid ninhydrin assay^68^. Initially, 0.1 g fresh leaves were ground in 5 mL 3% sulfosalicylic acid. The homogenate was centrifuged at 10,000 rpm for 3 min at 4 °C. The supernatant was mixed in a 1:1:1 ratio with glacial acetic acid, and 2.5% acid ninhydrin, boiled at 100 °C for 30 min and finally cooled. Then 6 ml of toluene was added, after thorough mixing, the chromophore-containing toluene was separated, absorbance was measured at 520 nm taking blank toluene as a control.

### Statistical analysis

One-way ANOVA followed by a Duncan’s multiple range test at a 5% level was used to statistically analyze the effect of Cd on plant growth, chlorophyll content, O_2_^·−^ production rate, H_2_O_2_ content, proline content, GSH levels, NPTs content, and antioxidant enzyme activity (APX, DHAR, MDHAR and GR). Each treatment was replicated three times. A Pearson correlation coefficient was calculated for each treatment to explore the relationship between O_2_^·−^ production rate, H_2_O_2_ content, proline content, GSH, NPTs content, APX, DHAR, MDHAR, and GR. A redundancy analysis (RDA) and a permutation test were performed for each treatment to determine the key variables explaining changes in the O_2_^·−^ production rate and H_2_O_2_ content. The predictor variables included proline content, GSH, NPTs content, APX, DHAR, MDHAR, and GR. A significance level of *P* < 0.05 was used in the Pearson correlation analysis, RDA, and Permutation tests. The Pearson correlation analysis, RDA, and permutation tests were carried out in R 3.5.2^69^. The ANOVA was carried out using SPSS software.

## Conclusion

In the current study, excessive Cd induces an increase of oxidants H_2_O_2_, O_2_^·−^, and the activities of APX, MDHAR, DHAR and GR were enhanced with exposure to increasing levels of Cd. Additionally, the AsA-GSH cycle was activated in honeysuckle in response to elevated Cd. According to the RAD and permutation tests, we confirmed proline and APX serve as the dominant antioxidant and antioxidant enzyme in scavenging ROS in elevated Cd. Collectively, GSH and NPTs also act as the secondary antioxidants and their levels increase in response to increasing concentration of Cd. In addition, the honeysuckle cutting growth was promoted by the addition of low concentrations (10 mg kg^−1^ and 30 mg kg^−1^) of Cd, but when honeysuckle cuttings were subjected to higher concentrations (150 mg kg^−1^ and 200 mg kg^−1^) of Cd, their capacity to tolerate Cd was exceeded and plant growth was inhibited. Due to honeysuckle's hyperaccumulator traits and relative resilience to moderate levels of Cd exposure, future studies should explore the genetic mechanism affecting Cd accumulation and determine the effectiveness of using honeysuckle as an active phytoremediation mechanism for areas contaminated with various levels of Cd.

## Supplementary Information

Supplementary Information.

## Data Availability

All data analysed during this study are included in this published article and its supplementary information files.
